# Evaluation of Biaxial Mechanical Properties of Aortic Media Based on the Lamellar Microstructure

**DOI:** 10.3390/ma8010302

**Published:** 2015-01-16

**Authors:** Hadi Taghizadeh, Mohammad Tafazzoli-Shadpour, Mohammad B. Shadmehr, Nasser Fatouraee

**Affiliations:** 1 Cardiovascular Engineering Laboratory, Faculty of Biomedical Engineering, Amirkabir University of Technology, 424 Hafez Ave., Tehran 15875-4413, Iran; E-Mails: h.taghizadeh@aut.ac.ir (H.T.); nasser@aut.ac.ir (N.F.); 2 Tracheal Diseases Research Center, National Research Institute of Tuberculosis and Lung Diseases (NRITLD), ShahidBeheshti University of Medical Sciences, Tehran 19575-154, Iran; E-Mail: mbshadmehr@sbmu.ac.ir

**Keywords:** aortic media, lamellar structure, microstructural modeling, strain energy function

## Abstract

Evaluation of the mechanical properties of arterial wall components is necessary for establishing a precise mechanical model applicable in various physiological and pathological conditions, such as remodeling. In this contribution, a new approach for the evaluation of the mechanical properties of aortic media accounting for the lamellar structure is proposed. We assumed aortic media to be composed of two sets of concentric layers, namely sheets of elastin (Layer I) and interstitial layers composed of mostly collagen bundles, fine elastic fibers and smooth muscle cells (Layer II). Biaxial mechanical tests were carried out on human thoracic aortic samples, and histological staining was performed to distinguish wall lamellae for determining the dimensions of the layers. A neo-Hookean strain energy function (SEF) for Layer I and a four-parameter exponential SEF for Layer II were allocated. Nonlinear regression was used to find the material parameters of the proposed microstructural model based on experimental data. The non-linear behavior of media layers confirmed the higher contribution of elastic tissue in lower strains and the gradual engagement of collagen fibers. The resulting model determines the nonlinear anisotropic behavior of aortic media through the lamellar microstructure and can be assistive in the study of wall remodeling due to alterations in lamellar structure during pathological conditions and aging.

## 1. Introduction

The arterial wall is functionally and structurally a complicated tissue. Successful biomechanical investigations of the aortic wall go back to only recent decades due to these complications [1]. The significance of such studies becomes multifold considering that pathological conditions of the cardiovascular system, such as coronary heart disease and hypertension, are among the leading causes of world mortality [2].

Since the mechanical properties of soft biological tissues highly depend on their microstructure, proposing a reliable mechanical model for these tissues, including the arterial wall, depends on the level of microstructure integration attained in the constitutive model. The early proposed models assumed the arterial wall to be a single continuous medium and suggested different forms of the strain energy function (SEF) to characterize its mechanical behavior [3,4]. These models contributed by providing primary insights into the mechanical features of arteries, but more realistic models with a focus on the microstructure were vital to fully understand the mechanisms involved in the mechanical behavior of arteries. As a consequence, a new category of arterial tissue models, called “microstructural models”, was adopted.

Microstructural modeling led to significant refinements, particularly in cardiovascular tissue modeling and recognizing how physiological and pathological states affect the state of arterial tissue [5,6]. In the case of the arterial wall, such models are classified into two categories. In the first category, arterial tissue is regarded to be composed of three main layers, *i.e.*, intima, media and adventitia [7,8]. The second category incorporates proposing different SEFs for the main wall fibrous constituents, such as elastin and collagen [9,10]. Since the mechanical behavior of the arterial wall highly depends on its fibrous lamellar structure, multiscale models have been recruited to relate the microstructural features of the arterial wall to its bulk mechanical behavior [11,12]. Homogenization techniques used in a multiscale approach are a powerful tool in modeling complex microstructures, such as collagen bundles and crosslinks within the arterial wall [13]. It is believed that elastic tissue contributes dominantly at low strains, while at higher strain ranges, collagen fibers become gradually engaged and their contribution ascends [14]. Although the active behavior of smooth muscle cells (SMCs) also contributes to the mechanical response of the tissue [15], their passive mechanical properties are negligible compared to those of elastin and collagen [16]. Since collagen fibers are three orders of magnitude stiffer than elastin [17], uncrimping of collagen fibers drastically stiffens the mechanical response of the tissue, leading to a nonlinear incremental stress-strain relationship for the entire tissue.

It is well known that the mechanical behavior of the arterial wall depends mainly on its composite-like lamellar structure and the mechanical properties of the media [18,19,20]. However, it should be noted that in supraphysiological loads, adventitia also contributes to the mechanical behavior of the arterial wall [5,14]. The lamellar organization of the media and its main building fractions have been delineated previously [21,22,23]. Wolinski and Glagov first uncovered the well-organized lamellar structure of arteries among different mammalian species. They observed alternating dark and light layers in stained slides of media and named them the “lamellar unit” of the media. The relationship between the numbers of these lamellar units (from six units in rat to 70 units in pig) and the physiologic pressure for the examined species revealed almost an equal force per lamellar unit among mammalian species [18]. Dingemans *et al.* provided a neat schematic representation of the lamellar unit of aortic media and depicted the organization of the extracellular matrix and the connection to smooth muscle cells (SMCs) [22]. O’Connel *et al.* provided a 3D schematic of the structure combining confocal and electron microscopy imaging, in which elastin sheets as elastic lamellae were distinguished from stiffer layers of collagen bundles and fine elastic fibers accompanied by SMCs [24]. Concentric elastin sheets are almost identical in terms of the thickness and also possess a nearly isotropic composition [18,25]. Within the interlamellar space, SMCs are located as the main organic constituent of media, with collagen and fine elastic fibers running between SMCs [22,24].

The incorporation of the histological and structural data of the arterial wall into mechanical models is crucial to inspect ongoing changes of the tissue from the healthy to diseased state, such as hypertension and also age-related wall remodeling. In this way, the material parameters of the model can be better correlated with the physical features of the tissue, and the alterations in these parameters define the respective alterations in the mechanics of the tissue.

Combining mechanical and histological data, it is convenient to inspect how the microstructure contributes to the mechanical characteristics of the wall among major arteries. Hence, the wall media can be considered as two sets of contiguous and concentric lamellae: sheets of elastin (Layer I) and interstitial layers consisting of collagen bundles and fine elastic fibers containing SMCs (Layer II).

To the best of our knowledge, the mechanical analysis of the lamellar structure of the media and its contribution to the whole wall mechanics have not been considered before; as a result, we have proposed a new approach to integrate the microstructure of the aortic media in a mechanical model. In our study, the pair of Layers I and II defines the “lamellar unit” of the media. Approximately 60 lamellar units form the aortic media in the thoracic region of an adult human aorta [18,23]. Utilizing biaxial mechanical data and geometric measurements of stained tissue rings, the material parameters of the proposed microstructural model are calculated, and the contributions of Layers I and II to the overall mechanical behavior of the media are explored.

## 2. Materials and Methods

### 2.1. Surgical Procedures

**Figure 1:**
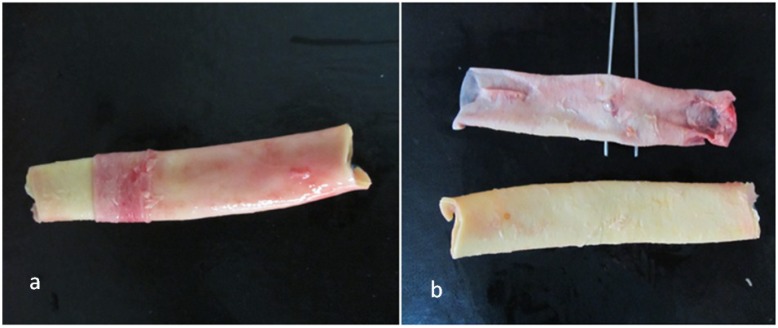
(**a**) Removal of adventitial tissue; (**b**) resulting intima-media composite and loose adventitial layer.

In the thoracic region of the descending aorta, the diameter changes are minimal, and the microstructural parameters, including the thickness and number of lamellae, do not vary notably. Hence, samples of human descending thoracic aorta were used. Arterial samples were provided from brain-dead patients after organ donation according to the Ethical Committee instructions of Masih Daneshvari Hospital, the main site of organ donation and transplantation in Iran. Aortic samples from three male donors, aged 25 (M25), 28 (M28) and 42 (M42) years, were used with special attention to the medical history of the donors. None of the subjects had shown a cardiovascular disorder and disease background. This is of particular relevance, since the mechanical properties of mature and healthy aortic media are addressed. Tubes of 5–6 cm in length were cut just above the diaphragm in the descending region of the aorta. Small branches were cropped carefully to achieve undamaged and intact tissues (Figure 1a). Sections from these samples were prepared for biaxial tests, and some adjacent blocks were extracted for histological staining.

### 2.2. Tissue Preparation

Aortic samples were preserved in phosphate-buffered saline (PBS) immediately after harvest and transferred to our lab. Before tests, the adventitial and loose connective tissues were carefully removed using a surgical scalpel (Figure 1). The remaining media-intima was submerged in PBS and then refrigerated at 4 °C for the test, preferably within the same day. Prior to tests, samples were allowed to reach room temperature.

Cylinders with a height of 11 mm were cut, and 11 mm × 11 mm squares were extracted for biaxial tests. Simultaneously, adjacent rings of 2–3 mm in height were cut to be used in histological staining.

### 2.3. Biaxial Testing

**Figure 2:**
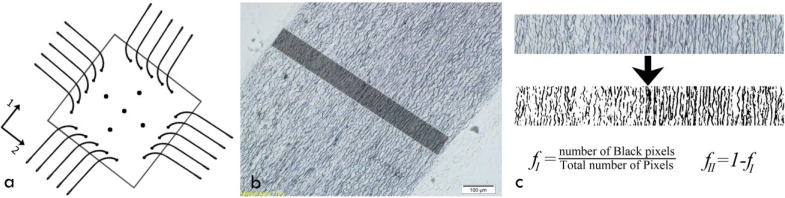
(**a**) A schematic of the sample under biaxial stretch; (**b**) Stained aortic media. Dark regions in this tissue section denote elastic fibers (Layer I), and light colored regions denote interlamellar zones, including collagen bundles, fine elastic fibers and smooth muscle cells (SMCs) (Layer II); (**c**) Volume fraction computation steps, including radial strip extraction, conversion to black and white and, finally, counting the number of pixels of the dark (Layer I) and light (Layer II) areas, as well as finding their proportion regarding the total number of pixels.

Biaxial testing of soft biological tissues is difficult, yet necessary, to fully comprehend the mechanical characteristics due to the complex microstructure and resulting anisotropic nature of the tissue. Such tests require a precise experimental setup, including the testing machine and attachment of the tissue to the jaws of the testing apparatus. Some researchers used uniaxial test data to characterize the mechanical properties [20,26]; others utilized a variety of biaxial tests, including planar biaxial tests [27] or extension-inflation tests [8]. In the present study, we carried out planar biaxial tests using a custom-made biaxial test apparatus consisting of four stepping motors to stretch the samples, while two load-cells recorded the load magnitude during extensions (Figure 2a). We used hooks to mount samples to the test machine. Such attachments are crucial to avoid unwanted stress concentrations. To record displacements, four markers were used on the central part of the square specimens, and a CCD camera was used to track the marker coordinates during the tests. The tissue thickness was measured several times with a caliper, and the average thickness was used in subsequent stress calculations. Considering homogeneous strain in the central region of the specimen, the resulting force-displacement data for both of the test axes were converted to second Piola stress and Green–Lagrange strain. The details of these conversions are provided in subsequent sections.

### 2.4. Histological Staining

In addition to biaxial test data, microstructural information, including dimensions and volume fractions of lamellae, is required in constitutive modeling. The volume fraction of each type of layer is based on the overall non-liquid phase, which contributes to the mechanical properties of the whole structure. These features were extracted by histological staining of extracted samples from human aorta. Sliced rings of aortic samples were first fixed in formalin, then paraffin-embedded and, finally, cut into micron-height tubes using a microtome [27]. The resulting slices were stained and investigated under light microscopy (Figure 2b). We used Verhoeff Van-Gieson (VVG) to stain elastin sheets (Layer I) to be distinguished from adjacent layers [27]. After staining, slides were photographed under a microscope, and the resultant images were processed by a MATLAB code to find the volume fractions. To do so, images were converted to black and white, and after removal of the artifacts, the percentage of the black (Layer I) and white (Layer II) zones were computed as the volume fractions. To minimize location dependency, we measured and averaged the volume fraction into six equally apart radial sites. Representative volume fraction computation steps are depicted in Figure 2c.

### 2.5. Constitutive Equations and Parameter Estimation

Experimental force-displacement data were converted to stress-strain. The resulting stress–strain response characterizes the mechanical behavior of the whole media. On the other hand, we assigned strain energy functions (SEFs) to Layers I and II and proposed the SEF of the media based on the SEFs of the layers. It should be noted that the SEFs of the layers are furnished with unknown material parameters, which should be identified. The SEF of the media was differentiated to give computational stress in terms of the mentioned material parameters. To evaluate these parameters, we compared experimental and computational stresses and minimized their differences by optimization of the material parameters through a nonlinear regression algorithm.

#### 2.5.1. Theoretical Framework

Describing the nonlinear and anisotropic response of the arterial wall requires utilization of continuum mechanics and hyperplastic models, due to the large deformations that arteries experience in the physiological environment [28]. Arteries are assumed to be incompressible, because of the high water content [29]. In a recent study, the degree of compressibility of different arteries has been shown to be small, especially for elastic arteries [30]. The SEF for such materials depends only on the deformation gradient *F*. Assuming that *X* and *x* denote the coordinates of the point in reference and deformed configurations, respectively, the deformation gradient is given by:

(1) $$F=\frac{\partial x}{\partial X}$$

Considering arteries as cylindrical tubes, it has been well described that axial, circumferential and radial directions coincide with the principal axes of arteries [31]. In the principal directions, *F* is represented by a diagonal matrix with components that are stretch ratios in the respective principal directions, Equation (2). Axial (λ_1_) and circumferential (λ_2_) stretch ratios are calculated from planar biaxial tests, and the radial component (λ_3_) is given by the incompressibility constraint ($J=\text{det}(F)=\lambda_{1}\lambda_{2}\lambda_{3}=1$):

(2) $$F=\left[\begin{array}{ccc} \lambda_{1} & 0 & 0\\ 0 & \lambda_{2} & 0\\ 0 & 0 & \frac{1}{\lambda_{1}\lambda_{2}} \end{array}\right]$$

SEFs are usually formulated as functions of left or right Green–Cauchy strain tensor ($B=FF^{T}$ and $C=F^{T}F$, respectively) invariants ($I_{1}$, $I_{2}$ and J) [32] or a Green–Lagrange strain tensor (E=12(C−I)) [31], which are interrelated functions of the deformation gradient. The second Piola stress for an incompressible material can be represented as [33]:

(3) $$S=-pC^{-1}+2\frac{\partial W}{\partial C}=-pC^{-1}+\frac{\partial W}{\partial E}$$

where *p* is a Lagrange multiplier term to enforce incompressibility and *W* describes SEF. The Lagrange multiplier parameter is determined from a boundary or pre-defined condition. In our case, the out of plane component of stress is zero (plane stress assumption), and the term *p* is obtained accordingly. After deriving the second Piola stress, other stress measures can be calculated and utilized accordingly. Cauchy stress is obtained by transforming the second Piola stress into a deformed configuration, which is given by:

(4) $$\sigma =\frac{1}{J}FSF^{T}$$

Experimental marker coordinates in biaxial tests were used to calculate respective stretch ratios, and subsequently, the deformation gradient tensor was formed. Then, the Green–Lagrange strain tensor ($E_{i}=\frac{1}{2}(\lambda_{i}^{2}-1)$, *E_i_* is used instead of *E_ii_* to denote diagonal elements of the Green–Lagrange strain tensor for simplicity) and the first invariant of right Green–Cauchy strain tensor ($I_{1}=\lambda_{1}^{2}+\lambda_{2}^{2}+\frac{1}{\lambda_{1}^{2}\lambda_{2}^{2}}$) were calculated for the time increments during the tests. Furthermore, the measured force per unit of the initial orthogonal cross-section of the tissue was calculated as the first Piola stress (*P*); then, it is converted to the second Piola stress:

(5) $$S=F^{-1}P$$

In the following sections, the deformation variables (Green–Lagrange strain and the first invariant of the right Green–Cauchy strain tensor) denote the experimental deformations. Parameter *S*_exp_ will denote the stress obtained from the experiments.

#### 2.5.2. Strain Energy Function

Appropriate types of SEF should be used for the media layers to evaluate the overall mechanical behavior of the tissue. To present proper forms of SEF for media layers, a phenomenological approach was adopted. It has been reported that the arterial elastin component is almost isotropic; hence, a neo-Hookean constitutive model can adequately elucidate its mechanical behavior [25,34,35]. Since, in this study, Layer I represents elastic lamellae, which are made almost entirely of elastin, neo-Hookean-type SEF was assigned to represent their mechanical behavior. Layer II, which is mechanically dominated by collagen fibers, behaves nonlinearly, due to the non-homogeneous distribution and gradual uncrimping of collagen fibers interwoven by fine elastic fibers and ground substances [36]. The four-parameter exponential SEF, which was proposed by Fung *et al.* [37], was used to simulate the mechanical performance of Layer II of the media. This function is capable of representing the nonlinear, anisotropic and stiffening behavior of the collagen-embedded biological materials and is described as “the most concise potential for biotissues” [38]. We assumed the superimposed contribution of Layers I and II, *i.e.*, Layers I and II act in parallel and contribute proportional to their volume fractions of the media. The superimposed contribution of components is widely used in arterial models [5,39]. Assigned forms of SEF to Layer I, Equation (6), Layer II, Equation (7), and the entire media, Equation (8), are discussed as follows:

(6) $$W_{\text{I}}=c_{1}(I_{1}-3)$$

(7) $$W_{\text{II}}=c_{2}[\text{exp}(a_{1}E_{1}^{2}+a_{2}E_{2}^{2}+2a_{3}E_{1}E_{2})-1]$$

(8) $$W_{\text{media}}=f_{\text{I}}W_{\text{I}}+f_{\text{II}}W_{\text{II}}$$

in which *f*_I_ and *f*_II_ represent volume fractions of Layers I and II of the overall non-liquid phase, respectively, and *W* denotes the SEF for Layer I (*W*_I_), Layer II (*W*_II_) and the whole media (*W*_media_). Additionally, *E_i_* stands for the Green–Lagrange strain and $I_{1}$ for the first invariant of the right Green–Cauchy strain tensor. Furthermore, *c*_1_, *c*_2_, *a*_1_, *a*_2_, *a*_3_ are unknown material parameters. Considering the concentric and parallel configurations of two layers and the fact that layers do not detach during stretch within the physiological range, it is assumed that the deformations of Layers I and II are equal to the deformation of the whole tissue, which is recorded during biaxial tests.

Bidirectional computational stresses for the whole media (*W*_media_) can be obtained in terms of layer stresses using Equations (3) and (8). Then, the following set of equations can be written for the computational axial ($S^{a}$) and circumferential ($S^{c}$) stresses of layers and the whole wall:

(9) $$\{\begin{array}{c} S_{\text{comp}}^{a}=f_{\text{I}}S_{\text{I}}^{a}+f_{\text{II}}S_{\text{II}}^{a}\\ S_{\text{comp}}^{c}=f_{\text{I}}S_{\text{I}}^{c}+f_{\text{II}}S_{\text{II}}^{c} \end{array}$$

The mechanical behavior of the media is expressed as a function of unknown material parameters proposed for the layers, Equations (6) and (7). The volume fractions of the layers were determined from image processing of the histological analysis, as described previously.

#### 2.5.3. Parameter Estimation

To find the unknown material parameters, the obtained experimental data were approximated with the proposed SEF in both circumferential and axial directions. Unknown material parameters accommodating computational stresses were estimated, such that computational stresses could best follow experimental stresses for the range of deformations (*E_i_*, *I*_1_). A MATLAB code was developed to search for the optimized set of unknown material parameters that can simultaneously best interpolate experimental data in circumferential and axial directions. Nonlinear regression was utilized to evaluate the error function as the squared difference of computational (*S*_comp_) and experimental (*S*_exp_) stresses in a full range of experimental deformations, Equation (10). The material parameters were updated in each iteration, till the minimum difference criterion was met.

(10) $$e(c_{1},\;c_{2},\;a_{1},\;a_{2},\;a_{3})=\sum_{n=1}^{k}\left[{\left(S_{\text{comp}}^{c}-S_{\text{exp}}^{c}\right)}_{n}^{2}+{\left(S_{\text{comp}}^{a}-S_{\text{exp}}^{a}\right)}_{n}^{2}\right]$$

Parameter *k* is used to summate the difference over the full range of deformations.

## 3. Results

**Figure 3:**
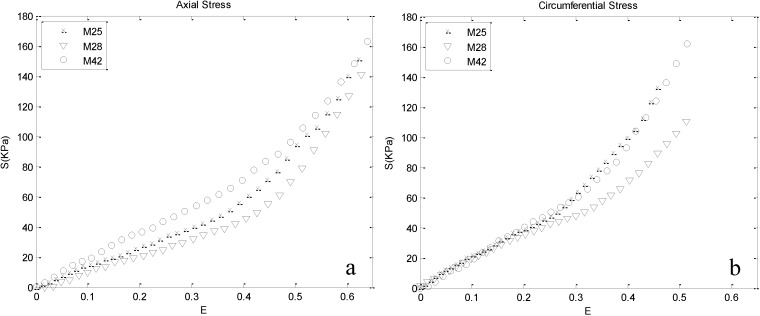
Stress-strain response obtained by biaxial tests: (**a**) axial and (**b**) circumferential responses. At low strain ranges, similar responses of the three cases in the axial and circumferential directions indicate that components leading to anisotropy do not contribute significantly in this region.

The nonlinear stress–strain responses calculated from the load-displacement data of human thoracic aorta obtained from biaxial tensile tests are depicted in Figure 3 for the axial and circumferential directions of all three test subjects. Comparing the respective strains, stiffer circumferential behavior is observed. The results indicate that in the initial part of the curves, the stress is proportionally increasing with the exerted strain up to a specific region, after which stress increases faster.

**Figure 4:**
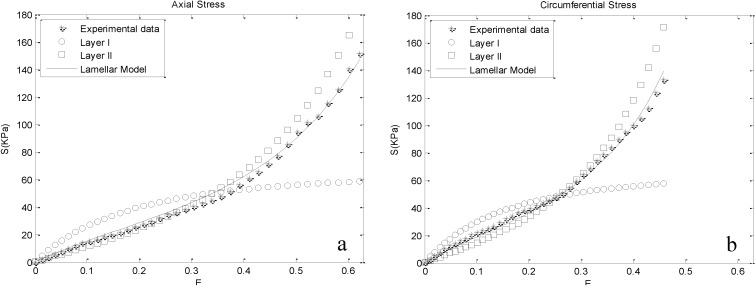
Experimental stresses plotted together with computational stresses for M25 (male donor, aged 25), based on the evaluated material parameters. The contribution of Layers I and II on the mechanical behavior of the media is depicted, as well, for the range of experimental strains: (**a**) axial direction; (**b**) circumferential direction.

These responses were fitted with the proposed lamellar model, and the resultant material parameters are delineated in Table 1. Goodness of fits were investigated for each case using the coefficient of determination (*R*^2^) and are reported in the same Table. The computational stress predicted by our model is plotted together with respective experimental data for one of the cases (M25) in Figure 4 to visualize the ability of the model to describe the anisotropic behavior of the aortic media. The same trend was reported for other cases. In addition, Figure 4 illustrates the contribution of Layers I and II on the stress-strain response of the media. In lower strains, Layer I bears higher stresses. By further straining, Layer II exceeds Layer I in stress. This pattern is observed in both the axial and circumferential directions. The intersection point of layer stresses denotes the equal contributions of them to the mechanical behavior of the whole media.

The computed volume fractions for Layers I and II utilizing image-processing are depicted in Table 1. For M25 and M28, the same range of volume fractions are found. However, for the third case (M42), representing a middle-aged subject, significant changes in the volume fractions are observed. For this case, the volume fraction of Layer II is drastically (~20%) elevated compared to younger subjects. This is in accordance with the effects of aging on the remodeling of the arterial wall due to new collagen synthesis, which is inherent within Layer II [40]. Moreover, it has been shown that aging is associated with decreased fibrillar crosslinks and changes in collagen fiber orientations in addition to altered collagen content [41,42]. Nevertheless, with such considerable changes in the microstructure, the stress levels of all three cases are similar.

**Table 1:** Computed volume fractions and obtained material parameters for the three cases tested.

Parameters	Cases		
	M25	M28	M42
*f*_I_ (-)	0.2803 ± 0.0113	0.2711 ± 0.0100	0.2172 ± 0.0067
*f*_II_ (-)	0.7197 ± 0.0113	0.7289 ± 0.0100	0.7828 ± 0.0067
*c*_1_ (kPa)	33.894	43.795	43.428
*c*_2_ (kPa)	35.877	15.692	46.627
*a*_1_ (-)	1.4655	1.9984	1.1188
*a*_2_ (-)	1.85712	2.2043	1.2865
*a*_3_ (-)	0.0473	0.0080	0.2265
*R*^2^ (-)	0.9946	0.9761	0.9959

## 4. Discussion

Distinct phases can be distinguished in the axial and circumferential stress-strain curves of the arterial wall tissue [20,43] (Figure 3). At low strains, the stress–strain curve is nearly linear and similar in both the axial and circumferential directions, indicating that components leading to anisotropic behavior of the artery wall do not contribute significantly in this range. The results of our lamellar model are in agreement with this fact, because at low strains, Layer I, which is assigned to arterial elastin, bears higher stresses compared to Layer II (Figure 4). The obtained material parameter for Layer I is consistent with recent investigations of the mechanical behavior of arterial elastin [25,44]. In the initial part of the stress–strain curve, collagen fibers behave in a wavy and crimped manner, and they remain almost inactive until further stretching, leading to their load-bearing engagement [45]. Along with further strain, collagen fibers gradually uncrimp and become engaged in the mechanical response, presumably described by the intersection of the stress-strain curves of Layers I and II (Figure 4). Further straining results in their activation, which is shown in the stress–strain curve of the media by a transition from a linear to a nonlinear response. Increasing stiffness of Layer II is owed to the gradual recruitment of collagen fibers. In this range of strain, Layers I and II contribute similarly to the mechanical behavior of the wall. The intersection point of the stress curves of the layers in Figure 4 denotes the equal contributions of Layers I and II. An interesting fact that can be inferred from Figure 4 is that the strain corresponding to the intersection point in the circumferential direction is lower compared to the axial direction, showing a stiffer circumferential response. This can indicate that more collagen fibers are aligned in the circumferential direction compared to the axial direction. In recent studies, the preferred direction of collagen fibers was inquired, and the same dominancy in circumferential direction has been reported [13,24,46].

Physiologic ranges of strains in aorta are reported to be maximally 36% in the axial and 21.5% in the circumferential direction [47]. Interestingly, these strains almost coincide with the intersection points in Figure 4, indicating almost equal layer stresses in physiologic strains. In the last phase, most of the collagen fibers are recruited [48], their behavior becomes dominant and the stress-strain response of the whole media follows the contribution of Layer II.

The measured volume fractions, besides providing geometrical data for the current lamellar model, can be used as an indicator of the structural changes in the lamellar structure of the media with age progression. It should be noted that for a comprehensive judgment, more samples of differently aged subjects should be investigated. Considering the drastic decrease observed in the volume fraction of Layer I in M42, together with the similar mechanical responses at low strains for all three cases, one can conclude that the elastin content of the media is mainly constant and increases in volume for Layer II, and the inherent deviations in the orientation and density of collagen fibers [42] are responsible for these changes. Unchanged elastin content and its decreased concentration in aging is well published [49].

The proposed microstructural model integrates the isotropic nature of arterial elastin, the anisotropic behavior of interstitial collagenous layers and their microstructural features simultaneously. Such models give more appropriate estimates of the mechanical response of the aortic media compared to conventional SEFs, such as the exponential function proposed by Fung *et al.* [37], since the proposed model in this study incorporates a separate term (the neo-Hookean function that is assigned to Layer I), which makes it more flexible to follow the nearly linear initial part of the stress-strain curve together with the exponential and anisotropic behavior of the tissue after the onset of collagen fiber activation (referred to as “biphasic behavior” in the literature [38]).

The lamellar model presented here is useful for establishing the roles of the micro-constituents of aortic media on the macro-behavior; this model is capable of following arterial mechanical behavior in its functional phases. The intended model provides some novel insights into the contributions of elastin and collagen to the mechanical behavior of the whole media. Further investigation is required in terms of the experiments and structural elements taken into account in the model. The mechanical properties of elastin sheets, collagen bundles, SMCs and also the interaction of these components are not fully understood, and novel experimental protocols are required. The accurate mechanical properties of these components along with realistic geometry will lead to more accurate models and new aspects of arterial mechanics.

## 5. Limitations

It was assumed that Layers I or II remain the same along the thickness of the media with the same mechanical properties. This assumption is adequate for describing the mechanical response of elastin sheets. However, as shown by advanced imaging techniques, such as second harmonic generation and multi-photon microscopy, collagen fibers change their orientation and possibly diameter as we move from the intimal side of the media towards the adventitia [22,50]. Since collagen is one of the main contributors to the mechanical properties of the media, a more accurate model that accounts for these alterations will lead to a better description of the mechanical properties. However, the proposed approach, which focuses on the lamellar microstructure of the media, is potentially capable of clarifying the underlying mechanics. Such models can describe the biomechanics of the arterial wall in health and disease, as well as the remodeling of the arterial wall due to aging or hypertension, based on the changes of the structural components.

## 6. Conclusions

In this study, a lamellar model of the arterial media based on both mechanical testing and microstructural information is proposed, and related material parameters are obtained. Samples of human thoracic aorta were examined biaxially to provide the mechanical data. Other required data, such as the volume fractions of layers, were computed from images of stained aortic rings using image processing techniques. Utilizing this new approach, some novel insights into the contribution of the media’s microstructure to the mechanical response are provided, which can be summarized as:

- Visualizing the contributions of Layers I and II for the range of physiologic and supraphysiological deformations.
- Providing an appropriate fit to biaxial test data of the aortic samples in both the axial and circumferential directions for different functional phases, *i.e.*, initial crimp and gradual activation of collagen fibers. The model predictions were in good agreement with the experimental data in both the circumferential and axial directions.

Compared to common microstructural models, the proposed approach in this study further investigates the mechanical contribution of lamellar units to the bulk of aortic media and determines how wall lamellae share functional loads. The proposed model in Equation (8) with the values of the material parameters reported in Table 1 can be regarded as a new framework of arterial models to investigate the physiological and pathological conditions of the arteries, such as aging.
